# Traditional Indian medicine and homeopathy for HIV/AIDS: a review of the literature

**DOI:** 10.1186/1742-6405-5-25

**Published:** 2008-12-22

**Authors:** M Fritts, CC Crawford, D Quibell, A Gupta, WB Jonas, I Coulter, SA Andrade

**Affiliations:** 1 Samueli Institute, 1737 King Street, Ste. 600 Alexandria, VA 22314, USA; 2 Johns Hopkins University School of Medicine, Center for Clinical Global Health Education, 600 North Wolfe Street, Jefferson 2-127 Baltimore, MD 21287, USA; 3 UCLA School of Dentistry, 63-037A CHS, 10833 Le Conte Ave, Los Angeles, CA 90095, USA; 4 Johns Hopkins University School of Medicine, Division of Infectious Diseases, 1830 East Monument Street, Ste. 8074 Baltimore, MD 21287, USA

## Abstract

**Background:**

Allopathic practitioners in India are outnumbered by practitioners of traditional Indian medicine and homeopathy (TIMH), which is used by up to two-thirds of its population to help meet primary health care needs, particularly in rural areas. India has an estimated 2.5 million HIV infected persons. However, little is known about TIMH use, safety or efficacy in HIV/AIDS management in India, which has one of the largest indigenous medical systems in the world. The purpose of this review was to assess the quality of peer-reviewed, published literature on TIMH for HIV/AIDS care and treatment.

**Results:**

Of 206 original articles reviewed, 21 laboratory studies, 17 clinical studies, and 6 previous reviews of the literature were identified that covered at least one system of TIMH, which includes Ayurveda, Unani medicine, Siddha medicine, homeopathy, yoga and naturopathy. Most studies examined either Ayurvedic or homeopathic treatments. Only 4 of these studies were randomized controlled trials, and only 10 were published in MEDLINE-indexed journals. Overall, the studies reported positive effects and even "cure" and reversal of HIV infection, but frequent methodological flaws call into question their internal and external validity. Common reasons for poor quality included small sample sizes, high drop-out rates, design flaws such as selection of inappropriate or weak outcome measures, flaws in statistical analysis, and reporting flaws such as lack of details on products and their standardization, poor or no description of randomization, and incomplete reporting of study results.

**Conclusion:**

This review exposes a broad gap between the widespread use of TIMH therapies for HIV/AIDS, and the dearth of high-quality data supporting their effectiveness and safety. In light of the suboptimal effectiveness of vaccines, barrier methods and behavior change strategies for prevention of HIV infection and the cost and side effects of antiretroviral therapy (ART) for its treatment, it is both important and urgent to develop and implement a rigorous research agenda to investigate the potential risks and benefits of TIMH and to identify its role in the management of HIV/AIDS and associated illnesses in India.

## Background

The Indian health system has perhaps the world's largest community-based indigenous system of medicine, and it includes Ayurveda, Unani medicine, Siddha medicine, yoga and naturopathy. [1] These forms of traditional Indian medicine along with homeopathy (hereafter abbreviated as TIMH) are commonly used. Reasons for TIMH use include a strong belief in TIMH efficacy as a "natural" and "holistic" option, and the fact that allopathic care is often costly, inaccessible and culturally dissonant. While the exact number of non-allopathic providers is not known as many are unregistered, India's Ministry of Health and Family Welfare has reported that there are over 700,000 registered practitioners of TIMH. [2] Over 65% of the population in rural areas of India are using TIMH and medicinal plants to help meet their primary health care needs.[3] TIMH is used to treat a wide variety of conditions, including cancer, diabetes and HIV/AIDS. [4-7]

### Overview of Systems of Traditional Indian Medicine

India has a long history of traditional medicine that is well established and integrated within the overall medical structure of the country. [8,9] In fact, there are more TIMH practitioners (over 700,000) [2] than allopathic medical doctors (approximately 633,000) in India, with Ayurvedic practitioners accounting for the largest number of TIMH providers. [10] Although TIMH use is prevalent in India, TIMH and non-TIMH practitioners still clash over issues involving these two domains of medicine. [11] TIMH practitioners are still labeled by some allopathic physicians as "quacks" who exploit Indian society by charging for TIMH practices that result in little benefit. [12] On the other hand, TIMH practitioners have not embraced the idea of testing their timeless remedies in clinical trials in order to meet the requirements of Western medicine. [11] This underscores the ongoing conflicts between TIMH and allopathic medicine in a country where TIMH is prevalent and supported by the India government.

To address some of these issues, the Indian government established the Department of Indian Systems of Medicine and Homeopathy in 1995 and later renamed it the Department of Ayurveda, Yoga-Naturopathy, Unani, Siddha and Homoeopathy (AYUSH), which is part of the Ministry of Health and Family Welfare. The mission of AYUSH includes: a) an initiative for integrating AYUSH with modern, allopathic medicine; b) attention to standardization of compounds and quality control; c) assessing and standardizing TIMH education in institutions around India that teach TIMH; d) improving the availability of raw material that will be used in the manufacturing of TIMH compounds; and e) prioritizing research on TIMH. The Indian Government has also established a Central Council for Research in each of these core areas, as well as separate Directorates of AYUSH in 18 Indian States. [13]

Not all TIMH is perceived equally in India. For example, yoga and healthy diet – which are often incorporated into the Ayurveda, yoga and naturopathy traditions – are considered beneficial by both TIMH and allopathic providers. However, many of the Ayurvedic herbal preparations and homeopathic treatments for conditions such as tuberculosis and HIV are discouraged by allopathic organizations such as the Indian Medical Association, or in allopathic medical schools, since data are lacking to support the use of such treatments. [10] Use of TIMH is documented in both urban and rural settings of India; however, the type of TIMH and the prevalence of use appear to vary by region and by rural versus urban milieu in India. [10]

#### Ayurveda

Ayurveda, which means "Science of Life," is a holistic medical system that emphasizes prevention and maintenance of health through creating balance of body, mind and spirit; self-awareness and self-care; and building harmony in relationships with others and the universe. Developed around 5,000 BC, many practices were passed on by word of mouth before the advent of written texts. [14] The *Caraka Samhita *and *Sushruta Samhita*, which are the primary texts on Ayurvedic medicine, describe eight branches of Ayurvedic medicine: internal medicine, surgery, treatment of head and neck disease, toxicology, psychiatry, sexual vitality, rejuvenation and care of the elderly, and gynecology, obstetrics and pediatrics. [15] Ayurvedic theory is based on three *doshas *(constitutional types), and diagnosis and treatment focus more on the individual's constitution (*prakriti*) than on the disease. Illness and other disorders are treated with combinations of herbs, oils, foods, yoga and lifestyle changes tailored to each person's constitution and designed to reduce symptoms, eliminate impurities, increase resistance to disease, and promote well-being. Ayurveda is the most frequently used system of TIMH. India has over 400,000 registered practitioners of Ayurveda, accounting for approximately 62% of its non-allopathic providers. In addition, there are over 2,000 Ayurvedic hospitals with nearly 44,000 beds, and over 200 Ayurvedic teaching institutions. [10]

#### Siddha medicine

The Siddha system of medicine has been practiced for over 5,000 years throughout southern India [6], and its development is attributed to 18 *Siddhars*, holy or "perfected" beings believed to have had superhuman powers. Traditional Siddha medicine is similar to Ayurveda in its identification of three *doshas*, and it focuses on prolongation of life through rejuvenating treatments and intense yoga practices, such as highly regulated breathing. [16] Mineral or metallic drugs are administered in very small quantities, and they are added to adjuvants (such as honey, ghee, milk, betel leaf juice and hot water), which are believed to modify the potency, toxicity and efficacy of the drugs. [17] Astrology and incantation are also an integral part of Siddha therapy. Use of Siddha medicine is most prevalent in Tamil Nadu, the southernmost state in India. In this state alone, there are over 100 Siddha hospitals, nearly 300 dispensaries, and over 11,000 practitioners. [13]

#### Unani medicine

The Unani system of medicine originated in Greece, was enriched by Arabic experts, and arrived in India during the medieval period. Unani theory is based on the tenet that balance among humors (blood, phlegm, yellow bile and black bile) is required for maintenance of health. Disease prevention and health promotion are achieved through emphasis on the "6 Essentials": pure air, food and water, physical movements and rest, psychic movement and rest, sleep and wakefulness, and retention of useful materials and evacuation of waste materials from the body. Unani treatments include medicines of herbal, animal, marine and mineral origin, as well as pharmacotherapy, diet therapy, and surgery. [13] There are over 42,000 registered Unani *hakims *practicing in India, and there are more than 250 Unani hospitals with over 5,000 beds. [10]

#### Homeopathy

The system of homeopathy came to India during the lifetime of its founder Dr. Samuel Hahnemann, a German physician who arrived around 1810 and treated patients, including Maharaja Ranjit Singh of Punjab. [13] Homeopathy aims to stimulate the body's natural defense mechanisms in order to prevent or treat illness. Treatment involves administration of very dilute doses of substances called "remedies" that would produce similar symptoms of illness in healthy people if they were given in larger doses. Treatment is individualized, and practitioners select remedies according to symptoms, lifestyle, and emotional and mental states. Based on Indian government data, homeopathic practitioners account for 29% of registered TIMH providers. [10] There are over 200 homeopathic teaching institutions and postgraduate departments in India.

#### Yoga

Developed in India over 5,000 years ago as a spiritual discipline, yoga is also used preventively and therapeutically. [18] Yoga practice is traditionally composed of physical postures, breathing exercises, meditation and relaxation. Variations of yoga practice have spread extensively throughout the West, where it is used primarily outside of medical settings. This interest has generated a number of Western scientific studies that have reported a variety of physiological and psychological benefits. [18,19] The Indian government does not collect data on the number of registered practitioners of therapeutic yoga, and it groups together research on yoga with naturopathy in its Central Council for Research on Yoga and Naturopathy. [13]

#### Naturopathy

The emphasis in naturopathy is on the patient as an integrated whole, and on cultivating wellness, prevention and self-care. The practice of naturopathy in India is divided into two approaches: one that is based in ancient Indian methods, and another that adopts primarily western methods such as physiotherapy. [13] The Western model of naturopathic medicine attempts to find the underlying cause of the patient's condition rather than focusing solely on symptomatic treatment, and its six fundamental principles are "the healing power of nature, trust in the body's inherent wisdom to heal itself, identify and treat the causes, first do no harm, doctor as teacher, and treat the whole person." [20] There are approximately 500 registered naturopathic practitioners in India, and just over a dozen hospitals with approximately 1,000 beds. [10]

### HIV/AIDS in India

With the completion of the National Family Health Survey III in 2006 and supplemental data from the National Behavioral Surveillance Survey and the Integrated Biological Behavioral Assessments Survey, India's National AIDS Control Organization (NACO) reduced the official burden of HIV infection to 2.5 million persons.[21] The epidemic is unevenly distributed across India, with six of India's 28 states accounting for approximately two-thirds of the estimated cases.[22] The 2006 estimates by NACO indicate that prevalence is highest among men (61% of all infections), the 15–49 age group (89%), and among high-risk subgroups such as Injecting Drug Users (IDUs, 9% of all infections), men who have sex with men (6%) and female sex workers (5%). The 2006 estimates indicate that the epidemic has stabilized in Tamil Nadu and other southern states but increased in the northern and eastern regions. Four of India's largest cities (Chennai, Delhi, Mumbai and Chandigarh) have a significant population living with HIV/AIDS, especially among IDUs. [23]

Of the estimated 785,000 people under 50 years of age, living with HIV and in need of ART, only 24,000 were reported by NACO to be receiving it in 2005. Access to these allopathic drugs has been increasing, primarily through the rapid expansion of ART delivery in government clinics; as of July 2007, over 100,000 patients were receiving ART in over 120 sites throughout India. [23] While the government scale-up in public hospitals and clinics now accounts for the majority of patients receiving ART, antiretroviral drugs are also provided through the private sector. [24] The complex medical health care seeking behaviors in India render many patients still in need of treatment. [10] For example, many patients prefer to pay out-of-pocket and receive care in private clinics, and lower-income patients often come to the public sector for hospitalized care when their resources have been expended. [25]

Little is known about TIMH use or its risk and benefits in HIV/AIDS management.[26] A survey of 1,667 HIV-infected persons in 4 regions of India found that 41% reported using some form of TIMH although only 5% believed TIMH was more effective than allopathic ART. [27] With many products prepared locally as well as available on the market and claims of "cure" being made, [28] there is a need for patients, providers and policy makers to assess systematically the potential benefit as well as potential harm associated with TIMH therapies for HIV/AIDS. Previous studies have shown that some natural medicines such as botanicals and herbal products can be potentially harmful to patients and thus, this is a research area of crucial importance that requires further investigation.

## Purpose

The purpose of this review was to survey and assess the quantity and quality of published, English-language literature on TIMH for HIV/AIDS treatment and care. Meta-analysis was neither intended nor possible due to the diversity of TIMH practices, therapies, and outcome measures in the extant literature. Quality scoring, such as assigning Jadad scores, [29] was not performed, since this was intended to be a descriptive review.

## Methods

To identify studies on HIV/AIDS and TIMH, MEDLINE, EMbase, BIOSIS, CINAHL, clinical trials.gov, the Cochrane Library and the National Library of Medicine catalog were searched using the following keyword sequence: [HIV and/or AIDS and India* and traditional medicine or ayurved* or unani or siddha or naturopathy or homeopathy or yoga]. Only studies in English were reviewed. Bibliographies of review and other relevant articles were searched for relevant Indian literature, as well as a bibliography of Indian medicine [30] and several easily-accessible Indian journals. A list of these additional journals is available upon request. Several reports and conference proceedings were also searched for relevant literature. Experts in each of the major systems of TIMH were contacted to request that they review the draft bibliography and identify additional key Indian literature in their fields of expertise. A list of these experts contacted is in the acknowledgments section.

### Study selection

We examined studies focused on the impact of TIMH on HIV/AIDS *in vivo*, *in vitro *and in clinical studies. Abstracts from the initial electronic searches were searched to select studies that met the following inclusion criteria: English-language, peer-reviewed literature, published after the first case reported of HIV in India in 1986 through October 2008. Randomized controlled trials, experimental studies, observational studies, descriptive studies, and reviews of HIV and TIMH were included, while ethnographic literature was excluded.

Two reviewers (MF and CC) independently screened the titles and abstracts of all of the literature collected through the searches. If both reviewers agreed that the abstract was relevant, the full article was accessed and a second phase of screening was conducted. All studies passing these screening phases were included in the full descriptive review.

## Results

Of 206 original articles reviewed, 36 were relevant and met the inclusion criteria described above. These 36 articles contained 38 relevant studies, 21 laboratory and 17 clinical. The majority of articles were excluded because they were not based on actual research studies, were not in English, or were surveys of TIMH usage. A bibliography of all included studies can be found in Additional File 1: Bibliography of Included Studies, and summaries of each of these studies can be found in Additional File 2: Summaries and Critiques of Included Studies.

### Review articles

A total of 6 previous reviews of the literature were identified that covered at least one system of TIMH. The review by Vermani and Garg [31] on TIMH for sexually transmitted diseases and AIDS was not exclusive to Ayurveda or to only HIV/AIDS, but it identifies many studies of Indian Ayurvedic herbs for HIV care, all of which we have included here. Ozsoy and Ernst [32] completed a systematic review on the effectiveness of complementary therapies for HIV/AIDS, which included only randomized, controlled studies on therapies such as herbal treatments, vitamins and other supplements, stress management and massage therapy. Mills et al. [33] reviewed complementary therapies for HIV treatment such as stress management, natural health products, massage, acupuncture, homeopathy and low-dose isopathy. Ullman [34] conducted a review on homeopathy for HIV care and identified five controlled clinical trials, three of which met our inclusion criteria. Martin and Ernst [35] completed a systematic review of antiviral agents derived from plants and herbs, but only one of the studies focused on HIV. Finally, Ernst's [36] systematic review of complementary AIDS therapies focused mainly on vitamins, massage, acupuncture and imagery, their prevalence of use, and treatment safety and costs.

This literature review identified 38 individual studies (21 laboratory studies and 17 clinical studies) that are relevant to TIMH and HIV/AIDS. Most studied Ayurvedic herbs and homeopathic preparations, and none of the articles reviewed addressed naturopathy or Unani medicine.

### Laboratory studies

Since the goal of this review was to assess the quality of published literature on TIMH for HIV/AIDS treatment and care, our main focus was on clinical research. However, 21 laboratory studies met the inclusion criteria and are summarized in Additional File 3: Table of Laboratory Studies. [37-57] Most of these studies involved cell lines, and the most commonly used was the H9 cell line. All reviewed studies examined Indian Ayurvedic herbs, and there were multiple studies on gossypol (*Gossypium spp*.), *Phyllanthus niruri*, curcumin (*Curcuma longa*), and neem (*Azadirachta indica*). Many studies used a controlled experimental model, with uninfected or mock infected cells as the controls. Almost all of these studies reported positive effects on HIV infection rates; however, many of these studies had significant methodological flaws, lacked information about product standardization, and had insufficient outcome measures and reporting.

### Clinical studies

Seventeen of the clinical research studies reviewed met the inclusion criteria. [6,57-70] A summary and critique of each of these clinical studies appears in Additional File 2: Summaries and Critiques of Included Studies, and Additional File 4: Table of Clinical Studies lists all studies included according to each system of TIMH. Most studies examined either Ayurvedic or homeopathic treatments for persons with HIV/AIDS. One study of Siddha medicine and one of yoga therapy were identified; no studies of naturopathic treatments for HIV/AIDS in India were found. Only four studies identified were randomized controlled trials; the others were pilot studies, case reports, observational and pre-post clinical studies. All of the studies were limited by small sample sizes (mean: 46 participants per study, range: 1–173 participants). Ten studies were published in MEDLINE-indexed journals. Overall, the studies reported positive effects, and some even suggested "cure," defined as seroconversion to HIV-negative status. Studies that reported effectiveness and improved outcomes include those of Ayurvedic and homeopathic treatments such as Boxwood (*Boxus sempervirens*), [67] Andrographolide (*Andrographis paniculata*), [62] and neem (*Azadirachta indica*), [57] as well as the Siddha combination therapy RAN (*Rasagandhi mezhuga, Amukkara chooranum *and *Nellikkai lehyam*).[6] The reader is referred to Additional File 2: Summaries and Critiques of Included Studies for a detailed summary and critique of each of these studies.

## Discussion

Overall, we found the methodological quality of published research on TIMH for HIV/AIDS to be poor, regardless of study design. General reasons for this poor methodological quality included lack of details on products and their standardization, small sample sizes, and high loss-to-follow-up rates. Design flaws included selection of inappropriate and/or weak outcome measures, uncertain representativeness of the study population, inadequate methods for determining exposure and outcome in observational studies, and short follow-up periods. Reporting flaws included incomplete reporting of study results, inadequately described withdrawals and dropouts, and reporting data only on those completing therapy. The four RCTs identified did not adequately report methods of randomization, blinding, withdrawals, and concealment of treatment allocation, as recommended by the Consolidated Standards of Reporting Trials (CONSORT) statement [71] that is aimed at the improvement of the quality of research reports of RCTs. Analytical flaws included weak handling of missing data, statistical analysis of data from non-completers using the last observation carried forward, and inadequate examination of the roles of patient characteristics, non-specific effects and other mediators, moderators and confounders of reported positive effects.

In sum, these methodological challenges and flaws in design, reporting and statistical analysis introduce bias and call into question the reviewed studies' internal and external validity. Claims of "cure" must be scrutinized since clinical and symptomatic diagnosis of AIDS is commonplace throughout much of the developing world, and establishment of an HIV-positive diagnosis through laboratory testing and routine confirmatory procedures (e.g., ELISA or Western Blot) is often cost-prohibitive.

### Limitations

This review may not represent all published literature on TIMH and HIV/AIDS, since neither the non-MEDLINE literature in India nor any other non-English literature was reviewed. Relevant unpublished articles and reports may have been missed, since the grey literature was not systematically searched. However, we suggest that searching the Indian literature would not have provided much added value for the following three reasons. First, we searched Indian Government reports on TIMH and HIV/AIDS and did not glean any additional important literature. Second, we spoke with several TIMH experts at a 2006 Research Agenda Conference on TIMH and contacted additional experts in each TIMH modality (see Acknowledgements section below), and these experts did not identify much additional important literature. Third, one author (IC) conducted a systematic review of Ayurvedic interventions for Diabetes Mellitus with colleagues at the RAND Corporation for the Agency for Healthcare Research and Quality, and the authors of this RAND review conducted a focused review of the Indian Ayurvedic literature and concluded that "limiting the language to English would decrease the literature yield, but we did not see evidence that it would significantly decrease the availability of studies most likely to be included in our review." [72]

### Research gaps

The Indian government has acknowledged that published data are sparse regarding the use, effectiveness and mechanism of action of TIMH in the treatment of HIV/AIDS. [1] Therefore, a pragmatic research agenda and concomitant funding are needed. As a first step in this process toward engaging the TIMH community and toward developing a research agenda, the Samueli Institute, Johns Hopkins University, the Indian Council of Medical Research and Seth Gordhandas Sunderdas Medical College/King Edward Memorial Hospital convened a research agenda conference on TIMH for HIV/AIDS in New Delhi in September 2006. Conference participants identified the following as priority areas: research training, infrastructure and methodology, effectiveness research, observational research/epidemiology, and product safety.

Additional areas where further research is needed are the impact of TIMH on quality of life, identification of TIMH therapies that could be used to treat HIV/AIDS-related complications, and identification of potentially immune-modulating TIMH compounds. Important safety-related challenges include interactions between ART and TIMH therapies for the management of HIV/AIDS, the unregulated Indian pharmaceutical industry, quality and purity issues, inadequate monitoring and standardization procedures, the presence of insecticides and heavy metals in TIMH treatments [73], and the availability of combinations of herbs over the counter that may interact with each other adversely and that may not be mentioned in ancient Ayurvedic texts. [74] Product-driven studies must characterize and standardize these compounds and then progress through *in vitro *and *in vivo *studies and phased clinical research culminating in methodologically sound pilot studies, and eventually larger-scale trials of TIMH.

Due to their methodological shortcomings, studies included in this review that reported positive effects [such as those of Boxwood (*Boxus sempervirens*), [67] Andrographolide (*Andrographis paniculata*), [62] neem (*Azadirachta indica*), [57] and the Siddha combination therapy RAN (*Rasagandhi mezhuga, Amukkara chooranum *and *Nellikkai lehyam*)] should be replicated to determine whether these initial positive effects can be confirmed and whether future research is justified. Future studies would benefit from larger samples, stronger designs, and clearer descriptions of populations, controls and intervention components under study. Imperative for reliability and validity is greater care in analysis and reporting of results.

### Challenges and proposed strategies to overcome them

Significant roadblocks to achieving these research goals exist. A broad spectrum of TIMH practices is currently in use, but there is limited availability of scientific information on which to build testable hypotheses. It is difficult to fit non-Western clinical practices that are often individualized for each patient into the Western, reductionist scientific model. Finally, there is a general lack of scientific expertise or a research culture within the TIMH practice community and a weak TIMH clinical perspective in the scientific community. The majority of TIMH research has been conducted outside the traditional setting in which the therapy was created and is practiced, which seriously limits the model validity and generalizability of research findings. [75] To overcome these roadblocks, both standard and innovative methodologies will be required, such as whole systems research, rapid ethnographic assessments and outcomes studies evaluating the use of TIMH as it is currently being practiced within India, and as an adjunct to allopathic care.

As previously stated, TIMH providers outnumber allopathic providers in India and serve as the primary method for delivering primary care for a majority of India's population. Across the socioeconomic spectrum, private medical facilities are the preferred source of care [76,77]. India's community-based and culturally relevant TIMH system is the predominant method of treating symptoms in rural and resource-poor settings and among patients who may not trust the allopathic system or find it culturally dissonant. Amidst a culture of mutual lack of knowledge, understanding, trust and recognition between the allopathic and TIMH systems, the recent use of TIMH therapies by allopathic providers and allopathic therapies (e.g., antibiotics) by TIMH providers is a cause for concern and action, since adverse drug-TIMH interactions have been identified and providers have not received the education necessary for safe administration of therapies outside of their area of training and expertise. [78]

While it would be imprudent to promote use of TIMH in place of allopathic medicines for HIV/AIDS, we propose that consideration be given by the Indian Medical Association to recognizing TIMH providers and to training them in providing coordinated care (in collaboration with their allopathic counterparts) to the millions of HIV infected patients for whom they already provide care. Furthermore, we propose that a sound, evidence-based model of integrative HIV/AIDS care that is based on enhanced cross-training and collaboration between TIMH and allopathic practitioners and solid safety and effectiveness data, has the potential for improving health outcomes among HIV positive individuals in India.

In summary, this review exposes a broad gap between the current widespread use of TIMH therapies for HIV/AIDS in India, and the research base supporting their effectiveness, efficacy and safety. The therapeutic effects of TIMH for HIV/AIDS cannot be established based on the current literature; most studies published to date in the English-language literature lack sufficient clarity and rigor of reporting to assess reliably and quantitatively the quality of results. The lack of high-quality evidence highlights the need for rigorous investigation of both whole systems of TIMH, as well as individual therapies. In light of the suboptimal effectiveness of vaccines, ART, barrier methods and behavior change strategies for prevention and cure of HIV infection, it is both important and urgent to develop a collaborative research agenda that uses rigorous methodologies to investigate, evaluate and better understand the role of TIMH in managing HIV/AIDS and associated illnesses in India.

## Competing interests

The authors declare that they have no competing interests.

## Authors' contributions

MF participated in the conception and design, acquisition of data, analysis and interpretation, drafting and critical revision of the manuscript, material support and supervision

CC participated in the conception and design, acquisition of data, analysis and interpretation, drafting and critical revision of the manuscript, material support and supervision

DQ participated in analysis and interpretation of data, drafting the manuscript, and material support.

WBJ participated in the conception and design, analysis and interpretation of data, critical revision of the manuscript, obtaining funding and supervision.

AG participated in the conception and design, analysis and interpretation of data, drafting of the manuscript, critical revision of the manuscript, administrative support and supervision.

AA participated in the conception and design, analysis and interpretation of data, critical revision of the manuscript, and supervision.

IC participated in the conception and design, analysis and interpretation of data, drafting of the manuscript, critical revision of the manuscript and supervision.

## Supplementary Material

Additional File 1 **Bibliography of included studies.** Click here for file

Additional File 2 **Summaries and critiques of included studies.** Click here for file

Additional File 3 **Table of laboratory studies.** Click here for file

Additional File 4 **Table of clinical studies.** Click here for file
