# Supplementary material for: Traditional Indian medicine and homeopathy for HIV/AIDS: a review of the literature
Source: AIDS Res Ther. 2008 Dec 22;5:25. doi: 10.1186/1742-6405-5-25 (PMC2637286; doi:10.1186/1742-6405-5-25)
Supplement: Additional File 1 — Bibliography of included studies. [file 1742-6405-5-25-S1.pdf]

Additional File 1: Bibliography of Included Studies

1. Barthelemy S, Vergnes L, Moynier M, et al. Curcumin and curcumin derivatives inhibit Tat-mediated transactivation of type 1 human immunodeficiency virus long terminal repeat. *Res Virol.* Jan-Feb 1998;149(1):43-52.

2. Bissuel F, Cotte L, Crapanne JB, et al. Trimethoprim-sulphamethoxazole rechallenge in 20 previously allergic HIV-infected patients after homeopathic. *Aids.* Apr 1995;9(4):407-408.

3. Brazier A, Mulkins A, Verhoef M. Evaluating a yogic breathing and meditation intervention for individuals living with HIV/AIDS. *Am J Health Promot.* Jan-Feb 2006;20(3):192-195.

4. Brewitt B, Traub M, Hangee-Bauer C, Patirck L, Standish L. Homeopathic growth factors as treatment for HIV: Recovery of homeostasis and functional immune system. Paper presented at: AIDS and Complementary and Alternative Medicine: Current Science and Practice, 2002; Philadelphia.

5. Brewitt B, Traub M, Hangee-Bauer C, Patrick L, Standish L. Homeopathic growth factors: a low cost survival strategy for functional immunity and improved metabolism. Paper presented at: XIIIth International AIDS conference; July 2000, 2000; Monduzzi Editore, Italy.

6. Calabrese C, Berman SH, Babish JG, et al. A phase I trial of andrographolide in HIV positive patients and normal volunteers. *Phytother Res.* Aug 2000;14(5):333-338.

7. Charmaine L, Menon T, Umamaheshwari K. Anticandidal activity of Azadirachta indica. *Indian J Pharmacology.* 2005;37:386-389.

8. Danninger T, Gallenberger K, Kraeling J. Immunologic changes in healthy probands and HIV infected patients after oral administration of Staphylococcus aureus 12c: a pilot study. *Br Homeopath J.* Jul 2000;89(3):106-115.

9. Danninger T, Gallenberger K, Kraeling J. Considerations relating to the epidemiology of human immunodeficiency virus infection: the impact of bacterial antigens and consequences for treatment. *J Altern Complement Med.* Apr 2003;9(2):299-309.

10. Deivanayagam CN, Krishnarajasekhar OR, Ravichandran N. Evaluation of Siddha medicare in HIV disease. *J Assoc Physicians India.* Mar 2001;49:390-391.

11. Durant J, Chantre P, Gonzalez G, et al. Efficacy and safety of Boxus sempervirens L preparations in HIV-infected asymptomatic patients: a munticenter randomized double-blind placebo controlled trial. *Phytomedicine.* 1998;5:1-10.

12. el-Mekkawy S, Meselhy MR, Kusumoto IT, et al. Inhibitory effects of Egyptian folk medicines on human immunodeficiency virus (HIV) reverse transcriptase. *Chem Pharm Bull (Tokyo).* Apr 1995;43(4):641-648.

13. Hansen J, Nielsen C, Nielsen C, et al. Correlation between carbohydrate structures on the envelope glycoprotein gp120 of HIV-1 and HIV-2 and syncytium inhibition with lectins. *Acquired Immunodeficiency Syndrome.* 1989;3(10):635-641.

14. Hu K, Kobayashi H, Dong A, Iwasaki S, Yao X. Antifungal, antimitotic and anti-HIV-1 agents from the roots of Wikstroemia indica. *Planta Med.* Aug 2000;66(6):564-567.

15. James J. Curcumin update: Could food spice be low-cost antiviral? *AIDS Treatment News.* 1993;176:1-3.

16. Kusumoto I, Nakabayashi T, Kida H, et al. Screening of various plant extracts used in ayurvedic medicine for inhibitory effects on human immunodefiency virus type 1 (HIV-1) protease. *Phytotherapy Res.* 1995;9:180-184.

17. Lee-Huang S, Kung HF, Huang PL, et al. A new class of anti-HIV agents: GAP31, DAPs 30 and 32. *FEBS Lett.* Oct 7 1991;291(1):139-144.

18. Li CJ, Zhang LJ, Dezube BJ, Crumpacker CS, Pardee AB. Three inhibitors of type 1 human immunodeficiency virus long terminal repeat-directed gene expression and virus replication. *Proc Natl Acad Sci U S A.* Mar 1 1993;90(5):1839-1842.

19. Lin TS, Schinazi R, Griffith BP, et al. Selective inhibition of human immunodeficiency virus type 1 replication by the (-) but not the (+) enantiomer of gossypol. *Antimicrob Agents Chemother.* Dec 1989;33(12):2149-2151.

20. Naik A, Juvekar A. Effect of alkaloidal extract of Phyllanthus niruri on HIV replication. *Ind J Med Sci.* 2003;57(9):387-393.

21. Nakane H, Ono K. Differential inhibitory effects of some catechin derivatives on the activities of human immunodeficiency virus reverse transcriptase and cellular deoxyribonucleic and ribonucleic acid polymerases. *Biochemistry.* 1990;29(11):2841-2845.

22. Ogata T, Higuchi H, Mochida S, et al. HIV-1 reverse transcriptase inhibitor from Phyllanthus niruri. *AIDS Res Hum Retroviruses.* Nov 1992;8(11):1937-1944.

23. Paice JA, Ferrans CE, Lashley FR, et al. Topical capsaicin in the management of HIV-associated peripheral neuropathy. *J Pain Symptom Manage.* Jan 2000;19(1):45-52.

24. Pharo A, Salvato P, Thompson C, et al. Evaluation of the safety and efficacy of SPV-30 (boxwood extract) in patients with HIV disease. *11th International AIDS conference, Vancouver, BC.* 1996;11(19):(abstract no. Mo.B. 180).

25. Polsky B, Segal SJ, Baron PA, et al. Inactivation of human immunodeficiency virus in vitro by gossypol. *Contraception.* Jun 1989;39(6):579-587.

26. Qian-Cutrone J, Huang S, Trimble J, et al. Niruriside, a new HIV REV/RRE binding inhibitor from Phyllanthus niruri. *J Nat Prod.* 1996;59:196-199.

27. Rastogi DP, Singh V, Dey SK, Rao P. Research studies in HIV infection with homoeopathic treatment. *CCRH Quarterly Bulletin.* 1993;15(3&4):1-6.

28. Rastogi DP, Singh VP, Singh V, Dey SK, Rao K. Homeopathy in HIV infection: a trial report of double-blind placebo controlled study. *Br Homeopath J.* Apr 1999;88(2):49-57.

29. Rimando AM, Pezzuto JM, Farnsworth NR, et al. New lignans from Anogeissus acuminata with HIV-1 reverse transcriptase inhibitory activity. *J Nat Prod.* Jul 1994;57(7):896-904.

30. Srikumar R, Parthasarathy NJ, Shankar EM, et al. Evaluation of the growth inhibitory activities of Triphala against common bacterial isolates from HIV infected patients. *Phytother Res.* May 2007;21(5):476-480.

31. Talwar GP, Raghuvanshi P, Mishra R, et al. Polyherbal formulations with wide spectrum antimicrobial activity against reproductive tract infections and sexually transmitted pathogens. *Am J Reprod Immunol.* Mar 2000;43(3):144-151.

32. Tharakan ST, Kuttan G, Kuttan R, et al. Immunostimulatory action of AC II--an ayurvedic formulation useful in HIV. *Indian J Exp Biol.* Jan 2008;46(1):47-51.

33. Turano A, Scura G, Caruso A, et al. Inhibitory effect of papaverine on HIV replication in vitro. *AIDS Res Hum Retroviruses.* Apr 1989;5(2):183-192.

34. Uckun FM, Chelstrom LM, Tuel-Ahlgren L, et al. TXU (anti-CD7)-pokeweed antiviral protein as a potent inhibitor of human immunodeficiency virus. *Antimicrob Agents Chemother.* Feb 1998;42(2):383-388.

35. Udeinya IJ, Mbah AU, Chijioke CP, Shu EN. An antimalarial extract from neem leaves is antiretroviral. *Trans R Soc Trop Med Hyg.* Jul 2004;98(7):435-437.
36. Usha PR, Naidu MU, Raju YS. Evaluation of the antiretroviral activity of a new polyherbal drug (Immu-25) in patients with HIV infection. *Drugs R D.* 2003;4(2):103-109.

Reviews Discussed

1. Ernst E. Complementary AIDS therapies: the good, the bad and the ugly. *Int J STD AIDS.* May 1997;8(5):281-285.
2. Martin KW, Ernst E. Antiviral agents from plants and herbs: a systematic review. *Antivir Ther.* Apr 2003;8(2):77-90.
3. Mills E, Wu P, Ernst E. Complementary therapies for the treatment of HIV: in search of the evidence. *Int J STD AIDS.* Jun 2005;16(6):395-403.
4. Ozsoy M, Ernst E. How effective are complementary therapies for HIV and AIDs?--A systematic review. *Int J STD AIDS.* Oct 1999;10(10):629-635.
5. Ullman D. Controlled clinical trials evaluating the homeopathic treatment of people with human immunodeficiency virus or acquired immune deficiency syndrome. *J Altern Complement Med.* Feb 2003;9(1):133-141.
6. Vermani K, Garg S. Herbal medicines for sexually transmitted diseases and AIDS. *J Ethnopharmacol.* Apr 2002;80(1):49-66.
