# Supplementary material for: Traditional Indian medicine and homeopathy for HIV/AIDS: a review of the literature
Source: AIDS Res Ther. 2008 Dec 22;5:25. doi: 10.1186/1742-6405-5-25 (PMC2637286; doi:10.1186/1742-6405-5-25)
Supplement: Additional File 2 — Summaries and critiques of included studies. [file 1742-6405-5-25-S2.pdf]

**Additional File 2**

**Summaries and Critiques of Included Studies**

**Contents:**


Ayurveda ..... 2
- James (1993) ..... 2
- Pharo, Thompson, and Mastman (1996) ..... 3
- Durant et al (1998) ..... 5
- Calabrese, Berman, Babish, et al (2000) ..... 8
- Paice, Ferrans, Lashley, Shott, Vizgirda, and Pitrak (2000) ..... 10
- Usha, Naidu, and Raju (2003) ..... 11
- Udeinya et al. (2004) ..... 13

Homeopathy ..... 14
- Rastogi, Singh, Dey, and Rao (1993) ..... 14
- Rastogi, Singh, Singh, Dey, Rao (1999) ..... 16
- Bissuel (1995) et al. ..... 18
- Brewitt (2002) ..... 19
- Danninger et al. (2003) ..... 20

Siddha ..... 21
- Deivanayagam (2001) ..... 21

Yoga ..... 23
- Brazier 2006 ..... 23

Naturopathy and Unani Medicine ..... 24

## AYURVEDA

### JAMES (1993)

#### SUMMARY:

James evaluated the possible benefit of curcumin, a chemical found in the food spice turmeric, for people with HIV. He makes short reference to a published study done by researchers at Harvard Medical School, which found curcumin as one of three inhibitors in the long terminal repeat (LTR) sequence of HIV. A limited comparison of AIDS trends in Trinidad among Indians (who eat much more curry containing turmeric and therefore curcumin) and Africans (who consume less curcumin) is made, and the paper states that Africans are 10 times more likely to have HIV but no reference to any other factors possibly contributing to this trend is made.

The article documents a case-study of one HIV-infected patient, who used turmeric extract, which contained about 100 times the concentration of curcumin than ordinary turmeric. The patient took three capsules three times a day (each containing 300mg turmeric extract concentrated and standardized for a minimum of preferred 95% curcumin in a base of whole turmeric). A week after taking the capsules, his regularly scheduled blood tests showed a significant drop in p24 antigen, a measure of viral activity.  The author makes no claim that curcumin has a use in treating AIDS. In fact, he states that the odds are against it. However, because of the availability and low expense of curcumin, he calls for additional research to be done on its possible positive benefits for people living with HIV/AIDS.

#### CRITIQUE:

The capsules were obtained from a health-food store, the dose was chosen randomly and safety was unknown. The results of this n of one study cannot be interpreted because 1) the dose used is not based on any *a priori* data, 2) the outcome measurement of p24 is not clinically relevant and 3) no reference

was made as to whether or not any other medication or supplement was being taken simultaneously. Little is known about the safety of curcumin and turmeric in large doses.

## PHARO, THOMPSON, AND MASTMAN (1996)

### SUMMARY

The authors evaluated the safety and efficacy of SPV-30, an all natural boxwood evergreen extract (manufactured by Arkopharma in France), in 173 patients infected with HIV. In all patients chosen for this study, CD4 and CD8 cell counts as well as HIV-1 plasma HIV RNA (RNA and branched DNA) were assessed. Each measure of immune function was evaluated at initiation, 2 months, 4 months, and 6 months. Every two months hematological and chemical parameters were assessed. At baseline, CD4 ranged from 0-860 cells/$mm^3$, CD8 from 42-3269 cells/$mm^3$, and plasma HIV-1 plasma HIV RNA ranged from undetectable to >2 million copies/ml.

Overall, 63% of patients experienced a decrease in plasma HIV RNA after six months, with 38% experiencing decreases of greater than 50% or 0.3 logs. For subjects with plasma HIV RNA >40,000 copies/ml, 66% experienced a decrease, with 35% having a decrease of over 50% or 0.3 log. The absolute CD4 cell count increased in 41% of participants (median change of 5 and 8.5% after 6 months), and 52% experienced increases in CD8 counts (median change of +22 or 2.1%). In participants with CD4 less than 200 cells/$mm^3$, 44% experienced increases (+2 or 14.3%) and in those with CD8 less than 500 cells/$mm^3$, 69% experienced an increase within 6 months (+76 and +31.5%).

No significant toxicities were noted and occasional side effects included mild diarrhea and abdominal cramping. Most participants noted improvement in diarrhea, energy levels, skin conditions, appetite, memory, concentration, sense of well-being, and weight gain. No significant abnormalities were observed with the hematological and chemical parameters. The researchers concluded that SPV-30

appears safe and effective as an adjunct therapy in treating patients with HIV, even in individuals at more advanced stages.

## CRITIQUE

There was no mention of any other details or demographic characteristics of the patients in the intervention or control groups.  There was no report of concomitant medications were being taken. The abstract did not mention the dosage amount or frequency of SPV-30 and adherence data to SPV-30 were not captured. This study had a number of design limitations. The authors did not provide a sample size calculation and thus, it is unclear whether the study was adequately powered. The study also lacked a control group making it challenging to objectively assess the effects of the intervention in the absence a comparative arm. The rationale for the selecting the dose of SPV-30 and the manufacture was not provided. Information about characterization and standardization for this compounded is also lacking. In addition, adherence to SPV-30 was not measured systematically during the study. Although the authors imply that SPV-30 is an effective therapy, it would have been informative to know that the patients took the intervention agent as prescribed. Many of the reported results were incomplete. For instance, the authors state that there were not significant abnormalities with the hematological and chemical laboratory parameters but numeric results were not provided in the abstract.

The lack of details also applies to the frequency of side effects and benefits of therapy with SPV-30 observed in this study.  There is also contradictory data since diarrhea is reported as both a side effect and a benefit of SPV-30 treatment. Although the authors inferred that there was a increase in CD4 cell count in subjects treated with SPV-30, the results are reported with negative signs preceding the numeric values implying a reduction and not an increase in CD4 cell parameters which is detrimental to patients with AIDS. Also, although the authors reported a reduction of 0.3 $\log_{10}$ in plasma HIV RNA after 6 months treatment with SPV-30, this seems a modest effect when compared to an expected HIV

plasma RNA decline of 0.75-1.0 $lg_{10}$ after a week of antiretroviral therapy. In summary, although it is reassuring to know that SPV-30 use did not result in significant toxicity, the many study design flaws could potentially had affected or biased the reported findings.

## DURANT ET AL (1998)

### SUMMARY:

The authors performed a randomized, double-blind, placebo-controlled, dose-comparison study to investigate the efficacy and safety of $SPV_{30}$, a preparation of Boxwood (*Buxus sempervirens* L.) in a total of 145 HIV-infected subjects. Inclusion criteria included: asymptomatic HIV, CD4 counts between 250 and 500 x $10^6$/l, platelet count >75 x$10^9$/l, hemoglobin >9.0mg/dl, serum transaminases less than five times the upper limit of normal values, serum creatinine <200mmol/l, Karnofsky score of at least 90%, and minimum of 18 years of age. Exclusion criteria included pregnancy, previous treatment with antiretroviral, immunomodulating or immunosuppressive agents, investigational drugs, systemic corticosteroids (>7days), and cancer chemotherapy. The authors performed a HPLC fingerprint analysis of SPV30 to characterize this compound.

At initiation, the patients were randomized into one of three groups: 1) $SPV_{30}$, 990mg/d (n=48) 2) $SPV_{30}$, 1980mg/d (n=49) or 3) placebo (n=48). Each patient took 2 capsules every 8 hours, and was evaluated every 4 weeks. Endpoints for participation (therapeutic failures) were defined as one of the following: 1) AIDS (defined according to the Revision of the CDC, AIDS Program, 1987); 2) AIDS-related complex (defined as oral candidiasis persisting despite treatment or relapsing after treatment, oral hairy leukoplakia persisting for 2 or more consecutive visits, multidermatome herpes zoster, weight loss exceeding 4.5 kg or 10% of body weight within a 6-month interval, or diarrhea defined as 3 or more liquid stools per day persisting for more than 30 days without an identifiable cause); 3) Decrease of CD4 count below 200 x $10^6$/l units.

The baseline demographics and biological characteristics of the treatment groups were similar at entry. The duration, originally set at 18 months, was shortened to approximately 37 weeks, as it was decided unethical to carry on this trial with a placebo group. Ten patients were lost to follow up, and 25 dropped out due to therapeutic failures. The study found a statistically significant reduction in therapeutic failures (decreases in CD4 count, patients progressing to AIDS or AIDS related complex) in patients taking $SPV_{30}$ 990mg/d (P=.036). Only three drop-outs were from the $SPV_{30}$ 990mg/d group compared to 12 from placebo and 10 from SPV 1980mg/d. The three that failed (from the 990 group) all had CD4 count <300 x $10^6$/l and plasma HIV RNA >100,000 copies/ml at baseline. An overall analysis of the plasma HIV RNA showed no statistical significant difference between groups. However, it was found that fewer patients, taking $SPV_{30}$ 990mg/d, had an increase of plasma HIV RNA greater than 0.5 log at the end of the study (P=.029). No severe side-effects were observed or reported in the three groups.

Researchers concluded that asymptomatic patients, with more than 350 x$10^6$/l CD4 and a relatively low plasma viral load have the opportunity to delay the rate of disease progression by taking $SPV_{30}$ 990mg/d. The investigators claimed that $SPV_{30}$ is highly safe, could be used as a complementary agent in the management of HIV asymptomatic patients, and call for further studies to confirm its efficacy.

## CRITIQUE:

Although this study had a sound design by using a randomized, double-blinded, placebo-controlled approach, it was interrupted prematurely by the DSMB. Thus, the intervention effect could only be quantified up to 37 weeks instead of the originally planned 18 month follow-up. Sample size justification was not provided, although the statistical plan for data analysis was available in detail. The rationale provided for study interruption was due to concerns that it was unethical to carry on the trial with a placebo group based on the results of the AIDS Clinical Trials Group (ACTG) study protocol number 175.

The ACTG 175 study evaluated mono and combination therapy with nucleoside analogues in HIV-infected persons and thus, it is unclear why the results from that trial would lead to stopping a study that was investigating a natural medication in patients not receiving antiretroviral therapy. In this study the authors made an effort to characterize the sample of SPB30 and also measured adherence. However, the methods for measuring adherence were not disclosed and the medication adherence was reported as good but numeric data was not provided.

Also, although the sample of SPV30 was characterized, it is unclear whether the SPV30 used in this study came from the same batch and whether there was extensive batch variability that could ultimately have influenced the study findings. As in the previous study by Pharo et al, the author reported no statistically significant differences between intervention and control regarding to adverse events but details about laboratory clinical parameters to support this statement were not provided. Also, the reason for a lesser effect with SVP30 dose of 1980 mg/d compared to a lower dose of 990 mg/d, is not clear. SPV30 is known to contain flavonoids which are chemical compounds reported to have antioxidant properties. Although the authors hypothesized that the higher dose of SPV30 would generate large amounts of flavonoids that in turn would trigger an oxidative stress, this inference conflicts with previous information provided in the manuscript that flavonoids found in SPV30 are known to have an antioxidant activity. Another significant challenge faced by studies investigating herbals is the lack of knowledge about their active compound(s) making it challenging for the design of future more target studies.

The authors concluded that SPV30 is safe and delays disease progression in patients without advanced AIDS and call for future studies in combination with antiretroviral therapy. However, before these studies are undertaken it will be important to establish that SPV30 does not alter the metabolism of antiretroviral agents.

## CALABRESE, BERMAN, BABISH, ET AL (2000)

### SUMMARY:

The authors assessed the safety and tolerability of orally administered andrographolide (from *Andrographis paniculata)*, as well as its effects on plasma HIV-1 RNA levels and on CD4+ lymphocyte levels, in a Phase 1 trial. The participants (13 HIV-positive patients and 5 healthy HIV-negative volunteers), male and female over the age of 18, and recruited from the Bastyr University Integrated Care Clinic and from the general population of Seattle, Washington. Exclusion criteria included pregnancy and detectable liver or renal abnormalities. Subjects were not taking antiretroviral therapy and were not using prophylactic antibiotics other than trimethoprim/sulfamethoxazole.

This was a dose escalating of PN355 (the major medicinally active component of *Andrographis paniculata*, a herb commonly used in China, India, and Thailand for the treatment of multiple diseases and ailments) beginning with 5mg/kg bodyweight, taken thrice daily for three weeks, followed by 10mg/kg bodyweight, thrice daily for the next three weeks, then 20mg/kg bodyweight, thrice daily for the last three weeks. The subjects were observed for three hours after the first dosage to detect any acute toxicity reactions, and then monitored closely for adverse events at weekly visits. Blood and urine specimens were also collected at entry and at weeks 3, 6, 9 for white blood cell count, chemistry, urinalysis, immunological (CD4, CD8, CD19 lymphocyte and NK cell levels) and virological assessment (plasma HIV RNA). Medication adherence was assessed by pill count and health status was evaluated using the Medical Outcomes Survey (HIV MOS). Subjects were contacted by phone between study to reinforce adherence, and to obtain information about adverse reactions and overall health status.

The doses studied were 5mg/kg bodyweight (weeks 1-3) and 10mg/kg bodyweight (weeks 4-6), and weekly observations continued into week 9 though no medication was taken after week 6. Intended for an nine-week duration, the trial was interrupted after 6 weeks because of a number of potentially

treatment-related adverse events. Patients continued to be followed off medications through week 9. Only one, HIV-negative participant did not complete the full nine weeks.

All but one (92%) of the HIV-positive patients reported at least one adverse event during the course of the trial, the most common were: headache, fatigue, rash, diarrhea, itchy hands and feet and a bitter/metallic taste in mouth. One HIV-positive subject experienced an anaphylactic reaction at week 4 of the study. 3 of the 5 HIV-negative subjects reported at least one adverse event, one dropping out due to a body rash. Despite the adverse events neither the HIV-infected or infected participants reported notable changes in the MOS scale. Plasma HIV RNA reduction was not statistically significant (p value not provided). Conversely, there was a significant increase in the CD4+ lymphocyte counts. The increase in HIV-positive individuals at week 6 was significant--after administration of 10mg/kg the CD4+ count rose from 405 cells/$mm^3$ to 501 cells/$mm^3$ (p=.002). However, in HIV-negative participants the CD4+ count unexplainably decreased (mean decrease of 147 and 196 cells/mm3 at weeks 3 and 6, respectively, compared mean baseline values) though there were no statistically significant changes (p=0.08), the researchers claim a larger trial is needed to assess the effects of andrographolide on plasma HIV-1 RNA levels.

The investigators concluded that andrographolide may inhibit HIV-induced cell cycle dysregulation, leading to a rise in CD4+ lymphocyte levels in HIV-1 infected individuals and suggested that due to the number of adverse events observed in this trial the dose of PN355 would need to be lowered from the starting dose of 5mg/kg bodyweight in future clinical trials.

## CRITIQUE:

Adherence results were not reported. In this abbreviated, small, well-designed Phase I dose escalating trial, the lack of a significant effect on plasma HIV RNA and the large number of adverse events, one of

them being rated as a serious allergic reaction, raises doubts about the future use of this agent in the management of patients with HIV/AIDS.

## PAICE, FERRANS, LASHLEY, SHOTT, VIZGIRDA, AND PITRAK (2000)

### SUMMARY

The authors studied topical capsaicin in the treatment of HIV-association distal symmetrical peripheral neuropathy (DSPN) in a multi-center, controlled, randomized, double-masked clinical trial. Twenty-six patients (25 men and 1 woman, 18 years or older with a mean age of 40.3) with HIV-related DSPN, identified from the rosters at two large urban hospitals, were enrolled in the study. Race, educational level, and employment status were assessed. Exclusion criteria were: lesions on the feet or legs, pregnancy, lactation, inability to read or speak English, use of dideoxyinosine or dideoxycytosine, or use of any topical medication on the lower extremities.

The subjects were randomized to receive topical capsaicin (0.075%) plus usual analgesic therapy (n=15) or the cream vehicle without capsaicin (placebo) plus usual analgesic therapy (n=11). Patients were instructed to apply the topical capsaicin cream (containing capsaicin (0.075%) in an emollient base) or the vehicle (containing only the emollient base without capsaicin) 4 times each day for 4 weeks and to record their pain score 4 times each day. A series of instruments were used to measure pain, pain relief, sensory perception, quality of life, mood, and function, such as The Brief Pain Inventory (BPI), the Quality of Life Index, the Profile of Mood States, and the Sickness Impact Profile.

At entry, current pain averaged 4.7 ± 2.6 and worst pain, 6.6 ± 1.8 on a 0-10 scale. There were no statistically significant differences at study entry between the capsaicin and vehicle groups with respect to age, education, CD4 count, time since HIV diagnosis, worst pain, current pain, or any of the scores measured by the instruments mentioned above.

The study found no improvement in measures of pain, pain relief, sensory perception, quality of life, mood or function when comparing the intervention and placebo groups. In fact, subjects who received capsaicin reported statistically significant increases in pain level (so severe that 5 dropped out of the study), which was expected, but was not followed by decreased pain in later weeks. The researchers, therefore, suggest that capsaicin is ineffective in relieving pain associated with HIV-associated DSPN.

### CRITIQUE:

Dropout rate was very high, with 12 subjects (46%) dropping out before the end of the 4-week study, and those receiving the capsaicin topical were much more likely to drop out (10 of the 12) than those receiving the vehicle. The study was limited by the low female enrollment (only one), the burning sensation reported by subjects which precluded complete double masking, and the self-report of information relating to analgesic drug use.

## USHA, NAIDU, AND RAJU (2003)

In an open-label pilot study, the authors evaluated the clinical efficacy and safety of Immu-25 in HIV-infected patients. This polyherbal contains extracts of immunoactive plants such as *Tinospora cordifolia* (100mg), *Withania somnifera* (100mg), *Emblia officinalis* (100mg) and *Ocimum sanctum* (100mg) and has been reported to protect nonspecific host defense mechanisms. The 36 patients (10 female, 26 male, mean age 35 years) chosen for the study were all HIV positive (confirmed by ELISA and Westernblot), were either asymptomatic or experiencing mild to moderate symptoms, and had a CD4 count <500 cells/µl. Patients taking ART , herbal preparations or immunomodulators agents, as well as patients with severe active infection, abnormal renal or hepatic function or total white blood count <2000 cells/mm$^3$ were excluded from the study.

Patients were given Immu-25 twice daily with 200 mL of water for 18 months. Patients also received an isoniazid and cotrimoxazole (sulfamethoxazole/trimethoprim) combination daily for prevention of

opportunistic infections . The patients were evaluated by the same investigator every 15 days for the first 4 weeks and then at 4-weekly intervals throughout the study. Vital signs, performance status and the severity and frequency of various HIV-indicative symptoms were evaluated using a 4-point scale. The presence of opportunistic infections was also evaluated using suitable radiological and serological tests. Lymphocyte phenotyping was done (using the flow cytometry technique) to evaluate the patient's immunological status, and routine hematological, biochemical, hepatic and renal laboratory parameters were checked before and every 6 months during therapy. Medication adherence was assessed by performing pill count.

The study showed a statistically significant increase in the patient's mean body weight from 58kg to 63kg after 6 months of treatment ($p<0.05$), and a further increase to 64kg after 12 months and 68kg after 18 months ($p<0.001$). Treatment produced a decrease in the mean plasma HIV RNA from 326,438 copies/mL (n=13) to 180,495 copies/mL (n=13) at 6 months and 22,069 copies/mL (n=9) at 12 months ($p=0.001$). There was a gradual increase in the mean CD4 cell counts from 243 cells/ l to 336 to 527 to 618 (at 6, 12, and 18 months). All were statistically significant at $p<.001$. Similarly, mean CD8 counts showed increases which were statistically significant ($p<.001$). There was a marked improvement in symptoms such as oral thrush, cough, and diarrhea, which started at 6 months (3.2 [2.7-3.7] baseline score to 2.1 [1.5-2.7] final) and fatigue and anorexia (which statistically significantly decreased after 12 months of treatment) from 3.6 (2.4-3.9) to 2.4 (1.2-2.8).

Medication adherence was high (>90% in 95% of the patients), and all patients tolerated the drug well with the exception of mild gastrointestinal symptoms. The overall effect of the herbal drug was rated as good or very good by 89% of the patients and by physicians in 87% of patients. The researchers suggest that Immu-25 has the potential to enhance the immune system of the patients with HIV/AIDS, effectively control various signs and symptoms of HIV, and elicits a good therapeutic response.

## CRITIQUE:

There was a high attrition rate; at the end of 18 months, 10 patients were lost to follow-up, and 14 of those who were followed up had not completed 18 months of treatment. The study is limited by the small sample size, lack of a placebo-treated control group, and the high drop-out rate. In addition, the authors did not provide a rationale for the selected dose of Immu-25 used in this trial.

# UDEINYA ET AL. (2004)

## SUMMARY

The authors evaluated an extract of neem leaf (*Azadirachta indica* A. Juss) for its antiretoriviral effects in lymphocytes and in a small group of patients with AIDS. The extract was also evaluated for its antimalarial effects in erythrocytes and its anticancer effects in endothelial cells. In the laboratory study, a 10 µg/mL acetone-water neem leaf extract conferred protection against HIV invasion in 75% of lymphocytes, compared to zero protection in the absence of neem by indirect immunofluorescence. In the clinical study, 10 patients with AIDS were given 1000 mg neem extract daily for 30 days. Included patients were antiretroviral- naïve, confirmed HIV-positive, had CD4 cell count <200 cells/µL, and had ≥1 AIDS-defining symptoms. Statistically significant improvements in hemoglobin level (9.8±2 before treatment and 12.1 ±1.5 after treatment; $p<0.05$), CD4+ cell count (126±45 before treatment and 241±60 after treatment; $p<0.01$) and erythrocyte sedimentation rate (90±32 before treatment and 49±28 after treatment; $p<0.01$) were reported, and improvements in body weight (57±8 before treatment and 60±9 after treatment; $p>0.05$) and platelet count (328±40 before treatment and 359±45 after treatment; $p>0.05$) were noted.

The authors reported no adverse effects and suggested that the mechanism of action may involve inhibition of cellular adhesion by HIV. The complete resolution of AIDS-related symptomatology during the treatment period is curious and may be an artifact of the very small sample size.

## CRITIQUE

Plasma HIV RNAs were not assessed, and the method of confirmation of HIV status was not stated. Nevertheless, these preliminary findings seem to warrant further study of the antiretroviral effects of neem in HIV-infected patients.

# HOMEOPATHY

## RASTOGI, SINGH, DEY, AND RAO (1993)

### SUMMARY

The authors conducted a four-year study (May1989-Dec1993) at the Regional Research Institute (RRI) for Homeopathy, Bombay to evaluate the effectiveness of homeopathic medicines and treatment in 190 patients with HIV/AIDS. The study objectives were to clinically control the progression of disease, enhance immune cell function through restoring normal CD4-CD8 ratio, observe seroconversion in HIV seropositive cases and to inhibit reverse transcriptase thereby blocking the replication of HIV.

HIV-infection was confirmed by ELISA and Western Blot. Participants were given counseling before and during treatment, and administered a selection of individualized homeopathic medicine. The selection of homeopathic regimen was based on totality of symptoms, constitutional features, hereditary influences and latest homeopathic softwares. However, the homeopathic therapies provide in this study was only reported for two participants. Clinical and serological measures, as well as AIDS-related symptomatology, were assessed periodically but details about frequency of assessment were not clearly provided. Exclusion criteria or use of concomitant medications are not mentioned in the manuscript. When any other health complications arose (diarrhea, fever, cough, oral candida, etc), the patients were treated with homeopathic medications accordingly. About 73.6% of the participants were CDC stage II (asymptomatic seropostive).

Hematological evaluation (n=93) showed improvement in total white blood cell (WBC) and granulocyte counts and hemoglobin levels in 17, 7 , 13 participants, respectively. The level of statistical significance of improvement for these laboratory parameters was not provided. The immunological profile (CD4 and CD8) was obtained in 94 participants, with follow-up tests in 27 of those cases. It is not clear why CD4 measurements were obtained at baseline and follow up in only a limited fraction of the participants. An improvement in CD4 cell count was reported in 21 (77.8%) of 27 participants and no change in 2 (7.4%). However, the absolute CD4 values were not provided. In the 124 under follow-up cases, the authors report  that 14 sero-converted and only 9 became symptomatic.

The authors provided more detailed information about the homeopathic treatment and response in three selected participants enrolled in this study. Large increases in body weight and sero-conversion were reported in two of the participants (both males, 27 years and 31 years. Case 3 was a male, 40 years, clinically classified under *Stage-IVA: AIDS Related Complex*, with bodyweight of 68kgs. “PHOSPHORUS” was selected and prescribed between January 1993 and December 1993 in different potencies (30, 200 in ascending order). Bodyweight was found to have increased to 74kgs, general condition of the patient is very good, no clinical abnormalities were found, and CD4/CD8 ratio moved from reversed to normal.  The investigators concluded that the use of homeopathic treatment in HIV-infected subjects improved the immune system, slowed down symptoms and progression to AIDS.

### CRITIQUE

This study is plagued by the small sample size, and the lack of a placebo comparator arm, statistical analysis, and detailed reporting of CD4 cell count data. Other methodological flaws include the lack of information about the use of concomitant medications that could potentially have affected the CD4 count and clinical response. In addition, the use of a variety of homeopathic remedies in the absence of information about standardization and characterization of these treatments makes the study findings

and the author’s conclusions difficult to interpret. What was meant by seroconversion is unclear since the authors stated upfront that all participants enrolled in this study were HIV-infected. The results are not interpretable because statistical comparisons were not made between the two groups. In addition, a sample size calculation was not provided and women and children were underrepresented.

## RASTOGI, SINGH, SINGH, DEY, RAO (1999)

### SUMMARY

Rastogi et al. evaluated the immuno-modulator role of homeopathic remedies in 100 HIV-positive patients (18-50 years, 71% male, confirmed by repeat ELISA and/or Western blot tests) in a 6-month randomized, double-blind, placebo-controlled clinical trial. Exclusion criteria included: past history of convulsions or cardiac disease and current need of medications to treat these conditions, zidovudine treatment four weeks prior to enrolment, and pregnant and lactating women.

Participants were stratified according to CD4 cell count and divided into two groups: (1) Asymptomatic HIV-infected (Center for Disease Control (CDC)-defined Stage II, n=50) and (2) HIV-infected with Persistent Generalized Lymphadenopahty (PGL; CDC-defined Stage III, n=50). In each group, 25 participants were randomly assigned to treatment and 25 to placebo.

There was not a significant difference in age distribution, body weight, immune status, or routine hematological values between the two groups at baseline. A single individualized homeopathic remedy was prescribed for each participant. A thorough homeopathic case was taken for each patient and individualized homeopathic potencies were used (25 different homeopathic remedies were prescribed at potencies of 6C, 30C, 200C, LM3, LM5, and the frequency ranged from one to three times daily). The methodology section did not include definitions about the homeopathic potencies terminology employed in this study. Processing and data analysis of symptoms were performed using computerized homeopathic software (HOMPATH®, Jawahar Shah, India). Data was captured using standardized forms.

Routine hematological investigations were carried out monthly and immunological and serological investigations every three months.

Eight of the asymptomatic (2 in placebo, 6 in treatment) subjects and twelve (7 in placebo, 5 in treatment) of the PGL group were lost to follow-up. In the PGL strata, the trial showed, within the treatment group, a statistically significant ($p<0.01$) increase in CD4 T-lymphocytes (433.30 [SD 195.19]and 534.35 [SD 278.23] before and after treatment, respectively), as well as a statistically significant elevation in CD8 T-lymphocytes (1234.20 [SD 539.77] and 1327.25 [534.68]; $p<.05$). The placebo group showed no significant increases in either parameter (433.61 [SD 152.48] 452.00] [SD 158.57] for CD4 cell count, and 1256.89 [SD 488.23] and 1298.50 [SD 293.97] for CD8 cell count, before and after treatment, respectively. Changes in body weight and other hematological parameters were not significant. Comparison among the control and the intervention arm did not reveal a significant p-value, though a clear difference between the means of CD4 (placebo=452, treatment=534) and CD8 cell counts (placebo=1298.5, treatment=1327.25) was observed. In the asymptomatic strata, differences in all outcomes, both within the two groups as well as between the groups, were not statistically significant.

The researchers called for future larger trials in this area in order to overcome the issue of high individual variations in immunological and hematological profiles. Acknowledging the ethical challenges of placebo trials in HIV-infected patients, the researchers called for carefully designed longitudinal and multi-centric studies, in various stages of the infection, to prove the efficacy of the therapy.

## CRITIQUE

Details about power and sample size calculation were not provided. A clearly and detailed data analysis plan was provided but details about power and sample size calculation were not found. Information about the placebo product used in this trial was lacking and details about its comparability in terms of

smell, color and taste with the homeopathic agents were unavailable.  Subjects with poor adherence, follow up less than 3 months, having taking any therapy, developed life threatening conditions or adverse effects of the therapy which require active interventions, and women who became pregnant in the study were considered lost to follow-up and not included in the final data analysis. Poor adherence and severity of side effects were not defined by the investigators.   A major limitation of the study was the small sample size and high loss to follow-up. Another weakness of the study is that the authors made no mention of whether or not other natural therapies were being used.

## BISSUEL (1995) ET AL.

### SUMMARY

The authors conducted a small (N=20) prospective study to examine the effectiveness of desensitization to trimethroprim- sulphamethoxamole (TMP-SMX) through a homeopathic dosing administration of this agent. This research is significant because nearly 80% of HIV-infected patients exhibit allergic reactions to TMP-SMX, which is used prophylactically for both *Pneumocystsis jerovenci (carinii)* pneumonia and toxoplasmosis.  TMP-SMX solutions were prepared using serial dilutions followed by succussion. Sucrose tablets were impregnated with both a 9 centesimal Hahnmemannian (cH) dilution and a 15 cH dilution. Following ten days of twice-daily sublingual administration of the 9 cH dilution and another ten days of the 15 cH dilution, 20 patients with a history of TMP-SMX skin reactions resumed TMP-SMX prophylaxis at doses of 80-400 mg/day. At follow-up (mean 6 months), 13 patients had responded well to homeopathic desensitization and had no adverse reactions, but seven did not tolerate TMP-SMX rechallenge.  There was no correlation with baseline CD4+ count. While nearly two-thirds of patients were successfully desensitized to TMP-SMX, more research is clearly needed.

### CRITIQUE

This small study lacked a control arm and blinding, and the range of follow-up was quite large (2-10 months).

## BREWITT (2002)

### SUMMARY

Brewitt conducted four studies to assess the use of homeopathic growth factors (hoGFs) as treatment for HIV infection. Growth factors are integral in the immune system aiding with immune responsiveness, wound healing, tissue repair and cell growth. Study A was a double-blind placebo design (n=21, 16 week duration) to assess the efficacy of homeopathic growth factors in patients with CD4 T cell counts of 130-570 cells/ml. Study B (n=55, 8 week duration, seven sites) was a test replication of study A. Study C (n=27, 36 weeks) was an open-label, follow-up study of available patients in Study A plus nine newly enrolled to evaluate the stability of the immune system. Exclusion criteria (for A, B, and C) included antiretroviral and corticoid steroid use or weight/LBM therapy. Only natural medicines and growth factors were allowed as treatment. Study D (n=26, 52 weeks) was an open label, follow-up study on studies A and C to evaluate survival and understand how hoGFs compared with AV therapies.

The investigators found a statistically significant weight gain in patients taking hoGFs in studies A and C. (p=.009 versus placebo at three months, p=.0004 versus placebo at four months). In Study B, after one month of treatment, a statistically significant difference in lean body mass change was found (p=.01). While increases in CD4 T-cells were reported in the hoGF groups, at the end of study D, CD4 counts were not statistically different than those of patients using AV therapy. Similar results were found for plasma HIV RNA. There was no statistical difference in plasma HIV RNA between homeopathic GFs and antiretrovirals. The researchers went on to conclude that hoGFs restore immune responsiveness and weaken HIV replication, suggesting it as a nontoxic, affordable, and effective treatment option for HIV-infected persons, especially those with low economic resources.

## CRITIQUE

There are several weaknesses in these studies. Very few statistical significance values were given, and clear definitions were greatly lacking throughout the study. Failure to provide information about what other natural medicines the patients were taking shows another weakness of the study. The author made no mention of what is included in “natural therapy” or the dosage or frequency of intake. Similarly, no specifics (particular medication chosen, frequency, dosage and rationale for its selection, standardization, characterization etc) of the antiretroviral therapy or hoGF therapy were outlined either. Medication adherence and reporting of adverse events or lack of them were not included in this report. These weaknesses could greatly limit the scope of the conclusions made.

# DANNINGER ET AL. (2003)

## SUMMARY

In a paper on the epidemiology of HIV infection, Danninger et al. (2003) report on studies of the effects of ultra-low doses of a lysate of *Staphylococcus aureus* Cown I (12c Potency). This study was undertaken based on the hypothesis that superantigens from organisms such as *Staphylococcus aureus* can cause permanent activation of the immune system leading to T-cell activation and consequently HIV replication. By administering the poison (in this case *Staphylococcus aureus* Cown I) in very small quantities, one can theoretically stimulate elimination of the same organism leading to a reduction in the antigen load to the body, with the intention of decreasing the frequency of the T-cell activation and viral replication. The authors justified their selection of *Staphylococcus aureus* Cown I, stating that this is one of the most ubiquitous strains of gram negative bacteria known to produce potent superantigens.

Twelve HIV- positive patients, 5 European (group I) and 7 African (group II), were treated for 1 year or 3 months, respectively. The outcome measures included circulating immune complex (CIC), B-lymphocytes, T-lymphocytes, CD4, CD8, CD4/CD8 ratio, time after the first administration. None of the

patients received antiretroviral therapy prior to the study and were advised to avoid other concomitant medications, vaccinations and other immune-stimulated procedures during the course of the trial. Patients were given an oral administration of *Staphylococcus aureus* Cown I in a 12c potency with 30% pure ethanol/water as solvent following the principals of the manufacturing of homeopathic medicines. Group I reported a decline in CIC measures for normal levels, clinical, CD4% and CD4/CD8 ratios improvements and remained stable over a period of one year. In one patient a sharp increase in CICs with clinical symptoms of rheumatic pains in arms and thighs was observed before declining to normal levels. Group II consisted of patients with severe immune deficiency. The average CD4% and CD4/CD8 ratio increased significantly from 10.2% (SD 6.9) to 26.0% (SD 7.5) and 0.23 (SD 0.23) to 0.49 (SD 0.25) , respectively, during the trial duration of 3 months.  Adverse events such as initial aggravation such rhinitis, bronchial secretion, fever, diarrhea, and skin eruption were observed in group I. However, these symptoms resolved spontaneously and no medical treatment was required.

### CRITIQUE

The demographics and socioeconomic characteristics of the patients were not mentioned nor was the dosage amount.

## SIDDHA

### DEIVANAYAGAM (2001)

### SUMMARY

Deivanagyam studied a series of case reports of 8 patients on a modified Siddha approach referred to as Varma. The treatment consisted of the patients having their legs smeared with special oil and immersed in ice for 30 minutes two times a day. Patients completed the study with 80 to 100 treatments. The results of the study showed that 2 patients had a reduction in their plasma HIV RNA with a CD4/CD8 increase. In one patient, plasma HIV RNA was below the level of detection and CD4 cell count increased

from 672 to 1,002 cells/mm3. The average weight gain was 2.5kg per month. There were not treatment withdrawals in this study. No adverse events were reported by the authors. The authors concluded that Siddha medicine in conjunction with OI drugs have a complementary role in improving clinical/ laboratory parameters in HIV patients.

A letter to a medical journal editor discussed the results of a controlled trial and a case series on Siddha therapies (Deivanayagam (2001). The first study tested the effectiveness of a formulation of 3 drugs (RAN) consisting of, rasagandhi mezhugu (RGM)[2], anukkara chooranam (AC)[3], and nellikkai lehyam (NL)[3], used to treat HIV infection. Sixteen patients with western blot/ immunoassay-proved HIV infection were divided in two groups. Eleven patients had RGM (500mg bid), AC (2.5g bid), and NL (5g/day) along with opportunistic infection (OI) controlling drugs (antituberculous therapy, clotrimoxazole for *Pneumocystis (carinii) jirovecii* pneumonia prophylaxis, Candida treatment and prophylaxis, and secondary prophylaxis of cryptococcal meningitis, The five remaining patients received only standard (OI) treatment. The authors reported that in the RAN group; 7 patients showed a reduction in plasma HIV RNA, an increase in CD4 cell counts, and an average weight gain of 2.2kg in 1 month. Also, six patients showed a mean reduction in plasma HIV RNA of 600,000 copies/ml. One patient withdrew from the study due to progressive central nervous system symptoms. The remaining three patients were reported as clinically stable without significant impact on plasma HIV RNA and CD4cell count. The control group gained an average of 0.75 kg in weight and showed an improvement in CD8 cell count. No patients in the control group experienced reduction in plasma HIV RNA. However, one patient who initially failed on the trial drugs was inducted into RAN and experienced a reduction in plasma HIV RNA.

## CRITIQUE

The demographics and socioeconomic characteristics of the patients were not mentioned. This trial had a number of limitations including the small patient sample, the lack of justification for the selected doses

of RAN, and no standardization or characterization protocols for the tested compounds, some of them known to contain up to 48 ingredients. Therefore, sound clinical trials are needed before RAN is either refuted or recommended as a potential treatment for patients with HIV/AIDS.

## YOGA

### BRAZIER 2006

#### SUMMARY

Brazier conducted a randomized control trial to evaluate the effectiveness of a group residential program aimed at improving the well-being of individuals with HIV infection. Sixty-two participants were recruited from community HIV/AIDS organizations in Vancouver, Canada. Participants were divided to two groups; an intervention group living at an Art of Living (AOL) residential facility in Quebec and those assigned to the control group remained to receive stand care in Vancouver. Measures such as the Mental Health Inventory, the Medical Outcomes Study (MOS)-HIV Health Survey, and the Daily Stress Inventory (DSI) were assed five times during the study and qualitative interviews were conducted between 4-6 weeks after the residential program ended.  ART consisted of 15 days in resident therapy including breathing techniques, meditation, movement, and group process. Twelve once a week follow-up sessions were administered to review the procedures learned during the program. Fifteen participants withdrew from the study due to conflicting schedules and not being able to adhere to the study requirements.

The authors reported that both groups were homogeneous and there were no significant differences between groups. Repeated measures analysis of variance indicated significant interactions between groups across time on the average MHI summary score (p=0.04) as well as other subscales: positive affect (p=0.03) and psychological wellbeing (p=0.02). The intervention and control group differed significantly on 3 of the 11 subscales of the MOS-HIV, including Social Function (p=0.001), General

Health (p=0.001), and Cognitive Function (p=0.02). Time effects occurred mainly at the 1st measurement point and after returning from the residential program. Overall group differences disappeared at the 6 and 12 week measurement points. The DSI indicated an increase in experience and impact of stress over time for the intervention group post-program. Fourteen participants agreed to be interviewed after program participation. The qualitative data concluded that the majority of those interviewed agreed that the program had a positive and profound change in their day-to-day lives.

### CRITIQUE

The demographics and socioeconomic characteristics of the patients were not reported in this study.

## NATUROPATHY AND UNANI MEDICINE

We found no published literature on naturopathy or Unani medicine that met our inclusion criteria.
